# Elevated Serum Levels of Soluble Transferrin Receptor Are Associated with an Increased Risk of Cardiovascular, Pulmonary, and Hematological Manifestations and a Decreased Risk of Neuropsychiatric Manifestations in Systemic Lupus Erythematosus Patients

**DOI:** 10.3390/ijms242417340

**Published:** 2023-12-11

**Authors:** Agnieszka Winikajtis-Burzyńska, Marek Brzosko, Hanna Przepiera-Będzak

**Affiliations:** 1 Individual Laboratory for Rheumatologic Diagnostics, Pomeranian Medical University in Szczecin, Unii Lubelskiej 1, 71-252 Szczecin, Poland; agawb@o2.pl; 2 Department of Rheumatology, Internal Medicine, Geriatrics and Clinical Immunology, Pomeranian Medical University in Szczecin, Unii Lubelskiej 1, 71-252 Szczecin, Poland; marek.brzosko@pum.edu.pl

**Keywords:** systemic lupus erythematosus, serum soluble transferrin receptor, interleukin 4, organ manifestations

## Abstract

The aim of this study was to analyze the relationship between the serum levels of soluble transferrin receptor (sTfR) and interleukin 4 (IL-4), and the disease activity and organ manifestations in SLE patients. We studied 200 SLE patients and 50 controls. We analyzed disease activity, organ involvement, serum sTfR, IL-4 and interleukin-6 (IL-6) levels, and antinuclear and antiphospholipid antibody profiles. The median serum levels of sTfR (*p* > 0.000001) and IL-4 (*p* < 0.00001) were higher in the study group than in the controls. SLE patients, compared to the controls, had significantly lower HGB levels (*p* < 0.0001), a lower iron concentration (*p* = 0.008), a lower value of total iron-binding capacity (TIBC) (*p* = 0.03), and lower counts of RBC (*p* = 0.004), HCT (*p* = 0.0004), PLT (*p* = 0.04), neutrophil (*p* = 0.04), and lymphocyte (*p* < 0.0001). Serum sTfR levels were negatively correlated with lymphocyte (*p* = 0.0005), HGB (*p* = 0.0001) and HCT (*p* = 0.008), and positively correlated with IL-4 (*p* = 0.01). Elevated serum sTfR > 2.14 mg/dL was associated with an increased risk of myocardial infarction (OR: 10.6 95 CI 2.71–464.78; *p* = 0.001), ischemic heart disease (OR: 3.25 95 CI 1.02–10.40; *p* = 0.04), lung manifestations (OR: 4.48 95 CI 1.44–13.94; *p* = 0.01), and hematological manifestations (OR: 2.07 95 CI 1.13–3.79; *p* = 0.01), and with a reduced risk of neuropsychiatric manifestations (OR: 0.42 95 CI 0.22–0.80; *p* = 0.008). Serum IL-4 was negatively correlated with CRP (*p* = 0.003), and elevated serum IL-4 levels > 0.17 mg/L were associated with a reduced risk of mucocutaneous manifestations (OR: 0.48 95 CI 0.26–0.90; *p* = 0.02). In SLE patients, elevated serum levels of sTfR were associated with an increased risk of cardiovascular, pulmonary, and hematological manifestations, and with a decreased risk of neuropsychiatric manifestations. In contrast, elevated serum IL-4 levels were associated with a decreased risk of mucocutaneous manifestations.

## 1. Introduction

Systemic lupus erythematosus (SLE) is a chronic autoimmune disease. The clinical picture of the disease varies from a mild course to a life-threatening disease [1]. In the course of the disease, skin, mucosal, hematological, musculoskeletal, cardiovascular, neurological, gastrointestinal, pulmonary, and renal organ manifestations may develop. In addition, SLE patients have an increased risk of accelerated atherosclerosis and cardiovascular complications, the development of which may be influenced by both the disease itself and the treatment used [1,2].

Hematologic disorders associated with abnormal iron metabolism are common in SLE. A lack of normal regulation in iron homeostasis can cause anemia of chronic disease (ACD) or iron deficiency anemia (IDA) [3]. Anemia in the course of SLE occurs in approximately 50% of patients [3,4]. The incidence of anemia is influenced by many factors such as inflammation, renal failure, gastrointestinal complications, and hemolysis. Numerous studies in SLE patients have reported that the prevalence of ACD ranges from 30% to 80% [4,5]. In many SLE patients, the differentiation of ACD and IDA is difficult. The determination of soluble transferrin receptor (sTfR), which is the plasma-soluble form of the transferrin receptor and an indicator of tissue iron deficiency, is helpful in differentiating between ACD and IDA. Elevated sTfR concentrations are indicative of an existing iron body deficiency or IDA. In ACD, sTfR concentrations are unchanged [6].

The dysregulation of iron homeostasis has been associated with several diseases including cardiovascular, neurodegenerative, depression, epilepsy, and respiratory tract diseases [7,8,9,10,11].

Interleukin 4 (IL-4) is an anti-inflammatory cytokine with a broad spectrum of effects. IL-4 stimulates the proliferation and differentiation of B lymphocytes and Th2 lymphocytes, inactivates the differentiation of Th1 lymphocytes and regulatory T cells (Treg), affects the production of IgE and IgG4, and is involved in granulopoiesis and erythropoiesis [12,13]. The role of IL-4 in SLE patients is ambiguous [13,14]. The available literature lacks a comprehensive analysis of the serum concentrations among sTfR and IL-4 and iron metabolism parameters, as well as organ manifestations in SLE patients.

The aim of this study was to analyze the relationship among serum sTfR and IL-4 levels, disease activity, and organ manifestations in SLE patients.

## 2. Results

The characteristics of the study group are shown in Table 1.

The median concentration of sTfR was higher in the study group than in the control group (*p* > 0.000001). The median serum IL-4 concentration was higher in the study group than in the control group (*p* < 0.00001) (Table 2).

The analysis of the hematological parameters showed that SLE patients, compared to the control group, had a significantly lower count of red blood cells (RBC) (*p* = 0.004), hematocrit (HCT) (*p* = 0.0004), platelets (PLT) (*p* = 0.04), neutrophil (*p* = 0.04), and lymphocytes (*p* < 0.0001), a lower HGB concentration (*p* < 0.0001), and significantly lower values of indices including the mean corpuscular hemoglobin (MCH) (*p* = 0.03) and mean corpuscular hemoglobin concentration (MCHC) (*p* < 0.0001). There was no significant difference between the study group and the control group regarding the white blood cell (WBC) count, the mean corpuscular volume (MCV) index value, and the number of reticulocytes (Ret) (all *p* > 0.05) (Table 2).

The analysis of the iron metabolism parameters showed that SLE patients, compared to the control group, had significantly lower iron (Fe) concentrations (*p* = 0.008) and lower total iron-binding capacity (TIBC) values (*p* = 0.03). There was no significant difference regarding the unsaturated iron-binding capacity (UIBC) value, nor in the ferritin, transferrin (Tf), and transferrin saturation (TfS) concentrations between the study and the control group (all *p* > 0.05) (Table 2).

The SLE patients showed a positive correlation between serum sTfR and IL-4 levels (*p* = 0.01). There was no significant correlation between sTfR levels and the patients’ age, disease duration, and IL-6 levels (all *p* > 0.05). 

In SLE patients, a negative correlation was found between serum sTfR levels and HGB levels (*p* = 0.0001), HCT (*p* = 0.008), MCV (*p* = 0.0001), MCH (*p* < 0.00001), and MCHC indexes (*p* < 0.00001), and the lymphocyte count (*p* = 0.0005). A positive correlation was found between the sTfR and WBC count (*p* = 0.03), Ret count (*p* = 0.001), and neutrophil count (*p* = 0.002). There was no significant correlation between the serum sTfR and RBC, PLT, monocytes, and eosinophils levels (all *p* > 0.05) (Table 3).

The study group showed a negative correlation between the serum sTfR and ferritin concentration (*p* < 0.00001), Fe concentration (*p* < 0.00001), and TfS index (*p* < 0.00001). In SLE patients, there was a positive correlation between the serum sTfR and Tf concentration (*p* = 0.001), TIBC (*p* = 0.007), and UIBC (*p* < 0.00001). There was no significant correlation between serum sTfR levels and CRP levels, fibrinogen levels, ESR values, and folic acid and vitamin B12 levels (all *p* > 0.05) (Table 3).

The comparison of SLE patients with and without different organ involvement, and with different severities of organ manifestations, showed elevated serum sTfR levels in SLE patients with myocardial infarction (*p* = 0.003), ischemic heart disease (*p* = 0.03), lung involvement (*p* = 0.02), and hematological manifestations (*p* = 0.002). SLE patients with heart lesions (*p* = 0.01), myocardial infarction (*p* < 0.00001), pericardial effusion (*p* = 0.01), and ischemic heart disease (*p* < 0.00001) were older compared to SLE patients without these lesions (Table 4). However, there was no correlation between the age of the SLE patients and the serum concentration of sTfR (R = −0.06, *p* = 0.3). Only one SLE patient had the coexistence of pericardial effusion with myocardial infarction and heart failure. 

A multivariate logistic regression analysis and stepwise analysis showed that in SLE patients, elevated serum sTfR > 2.14 mg/dL was associated with an increased risk of myocardial infarction (OR: 10.6 95 CI 2.71–464.78; *p* = 0.001), ischemic heart disease (OR: 3.25 95 CI 1.02–10.40; *p* = 0.04), lung involvement (OR: 4.48 95 CI 1.44–13.94; *p* = 0.01), and hematological manifestations (OR: 2.07 95 CI 1.13–3.79; *p* = 0.01), and with a reduced risk of neuropsychiatric manifestations (OR: 0.42 95 CI 0.22–0.80; *p* = 0.008) (Table 5). In SLE patients, there was no significant correlation between serum IL-4 levels and IL-6 levels (all *p* > 0.05). In the study group, there was no significant correlation between serum IL-4 levels and the patients’ age, disease duration, and the blood count parameters WBCs, neutrophils, lymphocytes, RBCs, HGB, HCT, MCV, MCH, MCHC, PLTs, and Ret (all *p* > 0.05) (Table 3). There were no significant correlations between serum IL-4 levels and other indicators of iron metabolism (all *p* > 0.05) (Table 3). No significant correlation was found between serum IL-4 levels and vitamin B12 or folic acid levels (all *p* > 0.05) (Table 3). In SLE patients, there was a negative correlation between serum IL-4 and CRP levels (*p* = 0.003) (Table 3). There was no significant correlation between serum IL-4 and fibrinogen levels or ESR values (all *p* > 0.05) (Table 3).

In a multivariate logistic regression analysis model and stepwise analysis, elevated serum IL-4 and elevated serum sTfR were not associated with the presence of antibodies in SLE patients (all *p* > 0.05).

The comparison of SLE patients with and without different organ involvement, and with different severities of organ manifestations, showed elevated serum IL-4 levels in SLE patients with mucocutaneous manifestations (*p* = 0.01) (Table 4).

In a multivariate logistic regression analysis model and stepwise analysis, elevated serum IL-4 levels > 0.17 mg/L in SLE patients were associated with a reduced risk of mucocutaneous manifestations (OR: 0.48 95 CI 0.26–0.90; *p* = 0.02) (Table 5).

## 3. Discussion

Based on our knowledge, our work represents the first comprehensive study of SLE patients, and we revealed that elevated serum sTfR levels are associated with an increased risk of cardiovascular, pulmonary, and haematological manifestations, and a decreased risk of neuropsychiatric manifestations. Additionally, elevated serum IL-4 levels showed an association with a reduced risk of skin and mucosal lesions.

Systemic lupus erythematosus is an autoimmune disease, during the course of which the occurrence of hematological and other organ manifestations is a significant clinical problem, and a variety of cytokines, such as IL-4, IL-6, and interleukin 10 (IL-10), can have a significant impact on this.

We conducted a study of SLE patients in whom serum levels of sTfR and IL-4 were evaluated in association with selected markers of disease activity, namely, hematological and other organ manifestations, iron metabolism parameters, and antibodies.

SLE patients have hematologic abnormalities which can either appear as an independent symptom or accompany other clinical manifestations [3]. Tomczyk-Socha et al. [15] compared the prevalence of hematologic manifestations in 71 SLE patients with short and long disease duration in a Caucasian population. They found the presence of hematological disorders in 53.5% of SLE patients at the time of diagnosis. In SLE patients with short and long disease duration, they found anemia in 33.8% and 42.3%, respectively, leukopenia in 32.4% and 33.8%, respectively, and thrombocytopenia in 18.3% and 12.7%, respectively. In another study of SLE patients from Turkey, the presence of hematologic symptoms was found in 67.3% of the subjects, of which AIHA was present in 6.5% and thrombocytopenia in 18.0% of the patients [16]. In SLE patients from Morocco, Zian et al. [17] found hematologic disorders in 46.0% of patients, including AIHA in 16.0%, lymphopenia in 30.0%, leukopenia in 8.0%, and thrombocytopenia in 8.0%. In our study, hematological symptoms were present in 69.5% of SLE patients, including AIHA in 8.5%, anemia of other types (ACD, IDA and ACD with IDA) in 43.4%, lymphopenia in 43.5%, leukopenia in 37.7%, and thrombocytopenia in 21.1%. These results are in agreement with data presented by other investigators [15,16,17]. This confirms the influence of iron metabolism disturbances on the development of hematological changes in SLE patients.

Soluble transferrin receptor is the plasma-soluble form of the transferrin receptor and is an indicator of tissue iron deficiency. Elevated sTfR concentrations are indicative of an existing iron deficiency or IDA. In ACD, the concentration of sTfR is unchanged [6]. In our study, we found significantly higher serum sTfR concentrations in the study group compared to the control group. Elevated serum sTfR levels in SLE patients may be indicative of impaired iron metabolism, suggesting an iron deficiency or increased erythropoiesis. In SLE patients, elevated serum sTfR levels may suggest the presence of IDA [18,19,20]. In our study, we demonstrated a positive correlation between serum sTfR levels and IL-4 levels in SLE patients. In the available literature, we did not find any studies showing a direct association between sTfR levels and IL-4 levels in SLE patients. In Kuvibidila et al.’s [21] study conducted on an animal model, it was found that serum IL-4 was positively correlated with Fe, the HGB concentration, and the HCT value. The hemoglobin level is an anemia marker with low-sensitivity and low-specificity, and it is unable to distinguish the type of anemia. HCT is an unreliable indicator in the diagnosis of anemia [19]. Reduced serum Fe levels stimulate sTfR synthesis. Our results suggest that IL-4 may have a stimulatory effect on the development of iron deficiency anemia in SLE patients.

IL-4 is a monomeric glycoprotein produced by Th2 lymphocytes, NK cells, mast cells, and basophils [22,23]. Interleukin 4 is an anti-inflammatory cytokine that inhibits the production of pro-inflammatory cytokines and acute-phase proteins such as haptoglobin, CRP, and albumin [20]. Observations of the results of IL-4 concentrations in SLE patients obtained by different investigators are divergent [13,24,25,26]. Zhou et al. [25] obtained comparable results regarding serum IL-4 concentrations in SLE patients and controls. On the other hand, Guimarães et al. [24] showed significantly reduced serum IL-4 concentrations in SLE patients compared to healthy subjects. In contrast, other researchers showed that IL-4 serum levels in SLE patients were significantly higher compared to the controls, which is in agreement with our results [15]. The discrepancy in these studies indicates that further research into the role of IL-4 in the pathogenesis of SLE is required.

Arora et al. [26] found a negative correlation between serum IL-4 levels and disease activity, as measured using the SLEDAI scale, and linked this to the anti-inflammatory and immunosuppressive effects of IL-4. In our study, we found no significant correlation between serum IL-4 levels and patient age, disease duration, and disease activity, as measured using the SLEDAI scale, but we demonstrated a negative correlation between serum IL-4 and CRP levels, confirming the anti-inflammatory effect of this cytokine in SLE patients. This confirms the inhibitory effect of IL-4 on disease activity in SLE patients.

In a study by Zhou et al. [25], it was shown that patients with positive anti-double-stranded DNA antibodies (anti-dsDNA) had lower IL-4 levels compared to patients with negative results regarding anti-dsDNA antibodies. Thus, serum IL-4 is likely to have an inhibitory effect on anti-dsDNA antibodies formation. In our study, no such relationship was confirmed. 

The determination of blood count and Iron metabolism parameters is crucial in diagnosing the type of anemia. Determining the type of anemia in SLE patients using conventional laboratory parameters is often difficult. The analysis of iron metabolism parameters in our study showed that SLE patients had significantly lower Fe and TIBC levels compared to the controls, which is in line with previous findings [4,27]. The results of a study conducted by Kunireddy et al. [4] in SLE patients showed significantly lower Fe, TIBC, and Tf levels compared to the controls, as well as elevated ferritin and hepcidin levels. In another study of SLE patients, elevated ferritin and reduced Tf and TIBC levels were observed compared to the controls [28]. In our study, there was no significant difference in the UIBC, Tf, or ferritin levels, nor in the TfS index between the study group and the control group. However, we showed that reduced ferritin levels were correlated with the risk of IDA, which is consistent with previous studies [4].

In the course of SLE, patients may develop lesions in multiple organs, but we do not have markers to predict that.

Iron disturbance is associated with abnormal cardiomyocyte function. Myocardial manifestations are most often accompanied by iron deficiency and/or anemia, which appear to be important factors contributing to a patient’s deterioration. Recently, sTfR levels were proposed as a potential new marker of iron metabolism in cardiovascular diseases. In the AtheroGene study, increased serum sTfR levels were strongly associated with future myocardial infarction and cardiovascular death [7]. In our study, when comparing SLE patients with and without different organ involvement, as well as varying severities of organ manifestations, we found that SLE patients with myocardial infarction and ischemic heart disease exhibited elevated serum sTfR levels. Additionally, we showed that elevated serum sTfR levels (>2.14 mg/dL) were associated with an increased risk of cardiovascular manifestations, such as myocardial infarction and ischemic heart disease, in patients with SLE. This confirms the influence of iron metabolism disturbances on the development of cardiovascular changes in SLE patients.

The mechanisms causing alterations in iron metabolism in the development of lung disorders are incompletely understood [8]. An increased accumulation of pulmonary iron is considered to play a key role in the pathogenesis of idiopathic pulmonary fibrosis and lung function decline [8,10]. In the available literature, we found no data on iron homeostasis dysregulation in SLE patients with pulmonary involvement. In our study, the comparison of SLE patients with and without different organ involvement, and with different severities of organ manifestations, showed elevated serum sTfR levels in SLE patients with lung lesions. Additionally, we demonstrated that elevated serum sTfR levels (>2.14 mg/dL) were associated with an increased risk of pulmonary manifestations in SLE. Therefore, we can conclude that in SLE patients, iron deficiency in conjunction with the autoimmune process may influence the occurrence of pulmonary lesions. Conducting further studies to identify the role of iron metabolism in the development of lung changes in SLE would be necessary.

Abnormal iron metabolism is associated with several neurological disorders. Iron deficiency anemia is associated with severe neurological impairments such as mental, neurophysiological, and emotional dysfunctions [11]. On the other hand, iron overload is one of the common causes of refractory epilepsy in patients with hemorrhagic stroke. However, the correlation between epilepsy and iron metabolism is not yet clarified and needs further exploration [10]. In our study, elevated serum sTfR levels (>2.14 mg/dL) were associated with a decreased risk of neuropsychiatric manifestations in SLE. However, we did not observe a significant difference in serum sTfR levels in the comparison of patients with and without neurological changes. In our opinion, these results confirm the ambiguous role of iron metabolism in the pathogenesis of neuropsychiatric changes in SLE patients. Further research on this problem is needed.

The results of Kalkan et al.’s [29] study suggested that there is a possible association between the functional IL4 VNTR genetic polymorphism and oral mucosal diseases of Turkish SLE patients. In our study, we showed that elevated serum IL-4 levels (>0.17 pg/mL) are associated with a reduced risk of skin and mucosal lesions in SLE patients. This confirmed the influence of IL-4 on the development of mucocutaneous changes in SLE patients.

Conducting further studies to identify the cytokines involved in organ manifestation may be helpful in personalized immunotherapy for SLE patients.

The fact that we found an association between sTfR levels and the occurrence of organ changes in SLE patients, without a correlation of this parameter with disease activity as measured by SLEDAI, may allow us to consider sTfR determination as a potential prognostic marker for the occurrence of selected organ changes in SLE patients. 

Our study’s strength lies in it highlighting the association of iron metabolism disturbances with the occurrence of organ manifestations in SLE patients. The control of iron metabolism markers, such as serum sTfR, may be helpful in assessing organ involvement and predicting disease progression. Additionally, this suggests the necessity to address iron metabolism disturbances as another treatment goal for SLE patients. The results of our study were not always in agreement with data from the literature, indicating that the mechanism of this relationship must be complex and requires further research. Therefore, a further analysis of these correlations will be the subject of our further research.

### Limitations

We acknowledge the limitations in our study due to the majority of patients having low SLEDAI scores. Consequently, we could not evaluate changes in the serum sTfR and IL-4 levels in patients with very low and very high disease activity, due to the small size of these groups. The majority of the patients were women, which is obvious because SLE occurs mainly in women. However, the limited number of male participants prohibits us from generalizing the obtained results to male SLE patients.

## 4. Materials and Methods

### 4.1. Patients and Controls

We studied 200 Caucasian patients with confirmed diagnoses of SLE and recorded data concerning their age, sex, disease duration, organ involvement, disease activity, and treatment. Recruitment of patients for the study took place during their routine visits to the outpatient clinic or at our rheumatology clinic. The control group consisted of 50 healthy individuals (44 females, 6 males), matched for age and sex with the study group and without data indicating organ changes. The ethics committee of the Pomeranian Medical University in Szczecin approved this study (KB-0012/11/13), and all participants provided informed consent.

The diagnosis of SLE was established according to the American College of Rheumatology (ACR) criteria of 1982 (modified in 1997) and the classifications developed by the Systemic Lupus International Collaborating Clinics (SLICC) of 2012 [30]. 

We assessed organ changes based on the SLIIC criteria, incorporating clinical, laboratory, and imaging data. Mucocutaneus manifestations encompassed the medical history of acute cutaneous lupus (malar rash and others), chronic cutaneous lupus (discoid rash and others), oral ulcerations, and nonscarring alopecia. Lung involvement was identified based on the presence of pleural effusions or pleural rub diagnosed using routine X-ray or computed tomography (CT). In addition, we documented data on nodular or interstitial lung lesions. Heart involvement was diagnosed based on the presence of typical pericardial pain lasting more than one day, pericardial effusion (confirmed through a two-dimensional echocardiography examination using a Philips Epiq 5 ultrasound machine), pericardial rub, or pericarditis observed in an electrocardiogram (ECG) (in the absence of other causes). Furthermore, we collected data on medical history related to hypertension, ischemic heart disease, and the number of myocardial infarctions. Renal lesions were diagnosed based on a 24 h urine protein output of 500 mg protein/24 h, or the presence of red blood cell casts. Neuropsychiatric involvement was considered based on clinical data of seizures, psychosis, mononeuritis multiplex (in the absence of other known causes such as primary vasculitis), myelitis, peripheral or cranial neuropathy (in the absence of other known causes such as primary vasculitis, infection, and diabetes mellitus), and acute confusional state (in the absence of other causes, including toxic/metabolic, uremia, drugs). Moreover, we collected data on medical history related to transient ischemic attacks and the number of strokes. Hematologic abnormalities were diagnosed based on assessments of blood morphology parameters, Coombs test results, and parameters of iron metabolism [30].

The disease activity of SLE was assessed according to the Systemic Lupus Erythematosus Disease Activity Index (SLEDAI) scale in a modified version: SLEDAI-2000 (SLEDAI-2K) [31]. 

### 4.2. Laboratory and Serological Diagnostics

For the estimation of sTfR, IL-4, and IL-6 levels, serum was stored at −80 °C until analysis using a sensitive sandwich enzyme-linked immunosorbent assay (ELISA) method using the Human sTfR Immunoassay Quantikine^®^ ELISA kit, Human IL-4 Immunoassay Quantikine^®^ ELISA kit, and the Human IL-6 Immunoassay Quantikine^®^ ELISA kit (R&D Systems, Minneapolis, MN, USA).

IgG antinuclear antibodies (ANA) were assessed in a HEp-2 cell line contaminated with CVCL-0030 cervical adenocarcinoma human HeLa using indirect immunofluorescence assay (IIFA). Monospecific tests were also performed using the ELISA method to detect anti-double-stranded DNA (anti-dsDNA), anti-Sm, anti-SS-A/Ro, anti-SS-B/La, anti-nucleosome (anti-NuA), anti-ribosomal P protein, anti-histone, and anti-U1-RNP antibodies (EUROIMMUN AG Medizinische Labordiagnostika tests, Lűbeck, Germany). The reference values of ANA were established as absent when the titer was <1:160 and present when the titer was ≥1:160. The titers were divided into three groups: low titers from 1:160 to 1:320, medium titers from 1:640 to 1:1280, and high titers > 1:1280.

The profiles of anti-phospholipid antibodies (aPL), including anticardiolipin (aCL) and anti-beta 2 glycoprotein I (aβ2-GPI), were determined using the ELISA method (EUROIMMUN AG Medizinische Labordiagnstika tests, Lűbeck, Germany). The lupus anticoagulant (LA) was tested using coagulological methods according to the criteria of the International Society of Thrombosis and Hemostasis [32].

Additionally, blood was taken for the assessment of ESR (Westergren method), C-reactive protein (CRP) (turbidimetric nephelometry), fibrinogen (Clauss method), and complement factors C3 and C4 (nephelometry) levels.

Blood count examination was performed using an automated method with XN-2000 and XN-550 hematology instruments from Sysmex (Kobe, Japan), using fluorescence flow cytometry (FFC).

The following blood morphology parameters were determined: hemoglobin (HGB), hematocrit (HCT), red blood cells (RBCs), white blood cells (WBCs), and blood platelets (PLT). The following blood cell indices were determined: mean corpuscular volume (MCV), mean corpuscular hemoglobin (MCH), mean corpuscular hemoglobin concentration (MCHC).

The following parameters of iron metabolism were determined: iron (Fe), ferritin, (Tf), transferrin saturation (TfS), total iron-binding capacity (TIBC), and unsaturated iron-binding capacity (UIBC), using COBAS 8000 (Roche Diagnostics, Mannheim, Germany). 

The vitamin B12 concentration and folic acid concentration were determined using the ECLIA method with COBAS 8000 (Roche Diagnostics, Mannheim, Germany).

### 4.3. Statistical Analysis

Data distributions were evaluated using the Kolmogorov–Smirnov test. Data are presented as means (SD) and medians (Q1, Q3). The R values of correlations were also determined. The groups were compared using a Student’s *t*-test, Mann–Whitney U test, and Kruskal–Wallis test. The parameters were evaluated using a Pearson’s chi-squared test (χ^2^), logistic regression analysis, and stepwise analysis, and *p* < 0.05 was considered statistically significant. All statistical data were analyzed using STATA 11: license number 30110532736 (StatSoft Inc., Tulsa, OK, USA).

## 5. Conclusions

In SLE patients, elevated serum levels of sTfR were associated with an increased risk of cardiovascular, hematological, and pulmonary manifestations, and a decreased risk of neuropsychiatric manifestations. In contrast, elevated IL-4 levels were associated with a decreased risk of mucocutaneous lesions.

## Figures and Tables

**Table 1:** Clinical characteristics of systemic lupus erythematosus patients and healthy controls.

Assessed Parameters		Study Group n = 200 Mean ± SD Number (%)	Control Group n = 50 Mean ± SD Number (%)	*p*
Sex		F: 181 (90.5); M: 19 (9.5)	F: 44 (88.0); M: 6 (12.0)	0.6
Age (years)		46.97 ± 13.73	42.72 ± 12.48	0.5
Disease duration (years)		10.40 ± 9.10	-	-
SLEDAI		10.07 (5.81)	-	-
Constitutional		52 (26.50)	-	-
Mucocutaneous			-	-
	Any change	135 (68.90)		
	Malar rash	115 (57.50)		
	Discoid rash	12 (6.00)		
	Oral ulcerations	44 (22.00)		
Arthritis		155 (77.50)	-	-
Heart		80 (40.4)	-	-
Myocardial infarction		10 (5.0)		
Ischemic heart disease		17 (9.6)	-	-
Hypertension		65 (32.5)	-	-
Lung			-	-
	Any change	12 (10.0)		
	Interstitial changes	6 (3.0)		
	Nodular lesions	4 (2.0)		
	Pleural effusion	2 (1.0)		
Haematologic involement				
	Any change	139 (69.50)	-	-
	Hemolytic anemia	10 (8.50)		
	Deficiency anemia	82 (43.40)		
	Leucopenia	75 (37.69)		
	Lymphopenia	87 (43.50)		
	Trombocytopenia	42 (21.11)		
Vascular system		29 (14.8)	-	-
Neuropsychiatric		68 (34.34)	-	-
Renal lupus		43 (21.50)	-	-
Treatment				
Antimalarials		154 (77)	-	-
Cs		162 (81)	-	-
Azathioprine		30 (15)	-	-
Cyclophosphamide		43 (21.5)	-	-
MMF		10 (5)	-	-
Methotrexate		7 (3.5)	-	-
Cyclosporin A		4 (2)	-	-
Immunoglobulins		12 (6)	-	-
Epratuzumab		2 (1)	-	-

n: number; F: female; M: men; SLEDAI: Systemic Lupus Erythematosus Activity Index; Cs: corticosteroids, MMF: mycophenolate mofetil.

**Table 2:** Laboratory characteristics of systemic lupus erythematosus patients and healthy controls.

Assessed Parameters	Study Group n = 200 Mean ± SD Median (Q1, Q3) Number (%)	Control Group n = 50 Mean ± SD Median (Q1, Q3) Number (%)	*p*
Sex	F: 181 (90.5); M: 19 (9.5)	F: 44 (88.0); M: 6 (12.0)	0.6
IL-4 (pg/mL)	0.00 (0.00, 1.58)	0.00 (0.00, 0.00)	<0.00001
sTfR [mg/L]	2.15 (1.6, 2.83)	1.51 (1.22, 2.04)	<0.00001
IL-6 (pg/mL)	2.50 (0.89, 5.40)	0.84 (0.30, 1.26)	<0.00001
ESR (mm/h)	16.00 (8.00, 30.00)	6.00 (4.00, 10.00)	<0.0001
CRP (mg/L)	1.89 (1.00, 5.83)	-	-
Complement factor C3 (mg/dL)	97.45 ± 25.2	-	-
Complement factor C4 (mg/dL)	16.86 ± 7.52	-	-
Fibrinogen (mg/dL)	349.5 ± 108.4	280.0 ± 65.5	0.0001
Positive direct Coombs test	25 (27.17)	-	-
False positive syphilis test (VDRL)	2 (1.90)	-	-
Hematological parameters			
WBCs (10^3^/µL)	5.71 (4.36, 7.46)	5.97 (4.73, 6.65)	0.7
Lymphocytes (10^3^/µL)	1.36 (0.99, 1.82)	1.81 (1.57, 2.17)	<0.0001
Neutrofils (10^3^/µL)	3.65 (2.55, 5.31)	3.33 ± 1.33	0.04
HGB (g/dL)	12.67 ± 1.70	13.85 ± 1.08	<0.0001
RBCs (mln/µL)	4.39 ± 0.53	4.65 ± 0.40	0.004
HGB (g/dL)	12.67 ± 1.70	13.85 ± 1.08	<0.0001
HCT (%)	38.05 ± 4.51	40.49 ± 3.03	0.0004
MCV (fl)	86.93 ± 6.55	87.32 ± 4.24	0.7
MCH (pg)	28.97 ± 2.74	29.87 ± 1.67	0.03
MCHC (g/dL)	33.26 ± 1.32	34.21 ± 0.89	<0.0001
PLTs (10^3^/µL)	227.4 ± 78.2	251.2 ± 55.6	0.04
Ret (‰)	10.00 (7.00, 14.00)	10.99 ± 5.07	0.5
Iron metabolism parameters			
Ferritin (ng/mL)	55.3 (23.6, 133.6)	43.1 (20.4, 109.6)	0.2
Tf (mg/dL)	260.0 ± 53.3	275.8 ± 54.6	0.06
Fe ug/dL	81.5 ± 46.2	100.7 ± 43.4	0.008
TIBC (ug/dL)	309.8 ± 64.6	331.0 ± 51.4	0.03
UIBC ug/dL	227.4 ± 84.0	230.3 ± 67.6	0.8
TfS (%)	27.40 ± 16.45	31.15 ± 13.01	0.14
Immunological assessment			
ANA IgG	198 (99.00)	1 (2)	-
Anti-dsDNA IgG	86 (45.70)	-	-
Anti-NuA IgG	57 (32.90)	-	-
Anti-Sm IgG	9 (5.10)	-	-
Anti-SS-A/Ro IgG	68 (39.10)	-	-
Anti-SS-B/La IgG	25(14.90)	-	-
Anti-SS-A/Ro IgG	68 (39.10)	-	-
Anti-SS-B/La IgG	25(14.90)	-	-
Anti-ARPA IgG	6 (3.50)	-	-
Anti-histones IgG	24(14.00)	-	-
Anti-U1-snRNP IgG	22 (12.60)	-	-
Anti-CL IgG	49 (28.00)	-	-
Anti-CL IgM	66 (37.70)	-	-
Anti-ß2-GPI screen IgA, IgG, IgM	61 (35.30)	-	-

IL-4: interleukin 4; IL-6: interleukin 6; sTfR: soluble transferrin receptor; ESR: erythrocyte sedimentation rate; CRP: C-reactive protein; WBCs: white blood cells; HGB: hemoglobin; RBCs: red blood cells; PLT: blood platelets; HCT: hematocrit; MCV: mean corpuscular volume; MCH: mean corpuscular hemoglobin; MCHC: mean corpuscular hemoglobin concentration; PLTs: platelets; Ret: reticulocytes; Tf: transferrin; Fe: iron; TIBC: total iron-binding capacity; UIBC: unsaturated iron-binding capacity; TfS: transferrin saturation; VDRL: Venereal Diseases Research Laboratory; ANA: anti-nuclear antibodies; Anti-dsDNA: anti-double-stranded DNA antibodies; Anti-NuA: anti-nucleosome antibodies; Anti-Sm: anti-Smith antibodies; Anti-SS-A/Ro: anti-Rose antibodies; Anti: ARPA: anti-ribosomal P protein antibodies; Anti-CL: anticardiolipin antibodies; Anti-B2GP-I: β2-glycoprotein I antibodies; Ig A: immunoglobulin A; Ig G: immunoglobulin G; Ig M: immunoglobulin M.

**Table 3:** The results of correlation analysis between sTfR and IL-4 levels and hematological, and iron metabolism parameters in patients with systemic lupus erythematosus.

Assessed Parameters	Levels of sTfR [mg/L]		Levels of IL-4 [pg/mL]	
	Spearman’s Rank Correlation Coefficient, R	*p*	Spearman’s Rank Correlation Coefficient, R	*p*
WBCs (tys/µL)	0.15	0.03	−0.11	0.1
RBCs (mln/µL)	0.00	1.0	−0.07	0.3
HGB (g/dL)	−0.28	0.0001	−0.04	0.6
HCT (%)	−0.19	0.008	−0.04	0.5
MCV (fl)	−0.28	0.0001	0.01	0.9
MCH (pg)	−0.36	<0.00001	−0.01	0.9
MCHC (g/dL)	−0.40	<0.00001	0.00	1.0
Ret (‰)	0.23	0.001	0.01	0.8
PLTs (tys/µL)	0.04	0.6	−0.06	0.4
Neutrofils (10^3^/µL)	0.21	0.002	−0.03	0.6
Lymphocytes (10^3^/µL)	−0.24	0.0005	−0.06	0.4
Monocytes (10^3^/µL)	0.02	0.8	0.04	0.6
Basophils (10^3^/µL)	−0.15	0.04	−0.01	0.9
Eosinophils (10^3^/µL)	−0.07	0.3	0.08	0.3
Ferritin (ng/mL)	−0.29	<0.00001	−0.04	0.5
Tf (mg/dL)	0.24	0.001	−0.09	0.2
TIBC (µg/dL)	0.24	0.0007	−0.09	0.2
Fe (µg/dL)	−0.39	<0.00001	−0.03	0.7
TfS (%)	−0.42	<0.00001	0.00	1.0
UIBC(µg/dL)	0.38	<0.00001	−0.05	0.5
sTfR (nmol/L)			0.17	0.01
Folic acid (ng/mL)	−0.02	0.8	−0.08	0.3
Vitamin B12 (pg/mL)	−0.07	0.3	0.00	1.0
CRP (mg/L)	0.08	0.3	−0.22	0.003
ESR (mm/h)	0.09	0.2	−0.07	0.3
SLEDAI	−0.02	0.7	−0.12	0.1

IL-4: interleukin 4; sTfR: soluble transferrin receptor; WBCs: white blood cells; RBCs: red blood cells; HGB: hemoglobin; HCT: hematocrit; MCV: mean corpuscular volume; MCH: mean corpuscular hemoglobin; MCHC: mean corpuscular hemoglobin concentration; Ret: reticulocytes; PLTs: blood platelets; Tf: transferrin; TIBC: total iron-binding capacity; Fe: iron; TfS: transferrin saturation; UIBC: unsaturated iron-binding capacity; CRP: C-reactive protein; ESR: erythrocyte sedimentation rate; SLEDAI: Systemic Lupus Erythematosus Activity Index.

**Table 4:** Comparison of serum sTfR and IL-4 levels in SLE patients with and without different organ involvement.

Organ Manifestations		Number of Patients	Age (Years)	*p*	Levels sTfR [mg/L]		Levels of IL-4 pg/mL	
					Median (Q1, Q3)	*p*	Median (Q1, Q3)	*p*
Mucocutaneous	-	61	46.43	0.5	30.34		0.34	
	+	135	47.36		28.34	0.12	0.00	0.01
	Malar rash	115	47.76	0.2	29.13	0.33	0.00	0.34
	Oral ulcerations	44	47.93	0.5	23.54	0.04	0.00	0.45
Heart	-	120	45.16	0.01	28.66	0.87	0.00	0.64
	+	80	49.98		29.45		0.00	
Myocardial infarction	-	173	45.96	<0.00001	28.62	0.0003	0.00	0.28
	+	10	67.20		43.02		0.00	
Number of myocardial infarctions	1	7	66.29	0.0002	51.29	0.0008	0.00	0.28
	>2	3	69.33		39.93		0.00	
Pericardial effusion	-	120	45.16	0.01	28.52	0.90	0.00	0.81
	+	73	49.98		29.45		0.00	
Ischemic heart disease	-	160	44.80	<0.00001	28.83	0.03	0.00	0.04
	+	17	66.82		38.04		0.00	
Lung	-	155	46.06	0.02	28.13	0.002	0.00	0.86
	+	12	56.92		36.82		0.00	
Type of lung lesions	Interstinal changes	6	46.43	0.08	33.44	0.46	0.00	0.75
	Other	6	52.75		34.01		0.00	
Haematological	-	107	47.48	0.09	23.71	0.002	0.00	0.66
	+	92	42.15		31.29		0.00	
Anemia	Haemolytic	10	37.50	0.05	32.40	0.003	0.00	0.84
	deficiency	82	47.33		18.27		0.00	
Thrombocytopenia	-	157	46.72	0.8	28.00	0.09	0.00	0.34
	+	42	47.64		32.02		0.00	
PLT (tys/µL)	<100	30	49.77	0.5	27.77	<0.00001	0.00	0.89
	≥100 <400	163	46.45		32.04		0.00	
	>400	7	47.14		61.13		0.00	
Leucopenia	-	124	48.02	0.1	28.67	0.37	0.00	0.54
	+	75	45.17		29.61		0.00	
Neuropsychiatric	-	131	46.66	0.5	30.27	0.27	0.00	0.56
	+	68	47.99		27.39		0.00	
Number of TIA or strokes	0	136	46.73	0.7	30.25	0.16	0.00	0.22
	1	51	48.22		23.71		0.00	
	>2	13	44.62		28.34		0.00	
Renal lupus	-	157	47.84	0.09	28.72	0.96	0.00	0.87
	+	43	43.79		30.29		0.00	

IL-4: interleukin 4; sTfR: soluble transferrin receptor; -: absence of organ involvement; +: presence of organ involvement; PLT: blood platelets; TIA: transient ischemic attack.

**Table 5:** A logistic regression model of the OR of the increased serum sTfR and IL-4 levels, and organ involvement in patients with systemic lupus erythematosus.

Organ Manifestations	Levels sTfR > 2.14 mg/L			Levels of IL-4 > 0.17 pg/mL		
	OR	95% CI	*p*	OR	95% CI	*p*
Constitutional	0.79	0.42–1.49	0.4	0.78	0.41–1.52	0.4
Mucocutaneous	0.74	0.40–1.35	0.3	0.48	0.26–0.90	0.02
Arthritis	0.74	0.38–1.42	0.3	0.76	0.39–1.47	0.4
Heart	1.14	0.65–2.02	0.6	1.04	0.58–1.86	0.8
Myocardial infarction	10.60	2.71–464.78	0.001	0.36	0.07–1.74	0.2
Ischemic heart disease	3.25	1.02–10.40	0.04	0.29	0.08–1.05	0.05
Hypertension	0.93	0.51–1.71	0.8	0.59	0.31–1.10	0.09
Lung	4.48	1.44–13.94	0.01	1.06	0.41–2.72	0.9
Haematological	2.07	1.13–3.79	0.01	0.96	0.53–1.77	0.9
Vascular system	1.47	0.66–3.26	0.3	1.34	0.61–2.97	0.4
Neuropsychiatric	0.42	0.22–0.80	0.008	0.59	0.31–1.13	0.1
Renal lupus	1.16	0.59–2.29	0.6	1.00	0.50–2.00	0.9

OR: Odds ratio, adjusted for gender and age; 95% CI: confidence interval; IL-4: interleukin 4; sTfR: soluble transferrin receptor.

## Data Availability

The data used to support the findings of this study are available from the corresponding author upon request.
